# Specific down-regulation of XIAP with RNA interference enhances the sensitivity of canine tumor cell-lines to TRAIL and doxorubicin

**DOI:** 10.1186/1476-4598-5-34

**Published:** 2006-09-05

**Authors:** Bart Spee, Martijn DB Jonkers, Brigitte Arends, Gerard R Rutteman, Jan Rothuizen, Louis C Penning

**Affiliations:** 1 Department of Clinical Sciences of Companion Animals, Faculty of Veterinary Medicine, Utrecht University, P.O. BOX 80.154, 3508 TD Utrecht, The Netherlands

## Abstract

**Background:**

Apoptosis resistance occurs in various tumors. The anti-apoptotic XIAP protein is responsible for inhibiting apoptosis by reducing caspase-3 activation. Our aim is to evaluate whether RNA inhibition against XIAP increases the sensitivity of canine cell-lines for chemotherapeutics such as TRAIL and doxorubicin. We used small interfering RNA's (siRNA) directed against XIAP in three cell-lines derived from bile-duct epithelia (BDE), mammary carcinoma (P114), and osteosarcoma (D17). These cell-lines represent frequently occurring canine cancers and are highly comparable to their human counterparts. XIAP down-regulation was measured by means of quantitative PCR (Q-PCR) and Western blotting. The XIAP depleted cells were treated with a serial dilution of TRAIL or doxorubicin and compared to mock- and nonsense-treated controls. Viability was measured with a MTT assay.

**Results:**

All XIAP siRNA treated cell-lines showed a mRNA down-regulation over 80 percent. Western blot analysis confirmed mRNA measurements. No compensatory effect of IAP family members was seen in XIAP depleted cells. The sensitivity of XIAP depleted cells for TRAIL was highest in BDE cells with an increase in the ED_50 _of 14-fold, compared to mock- and nonsense-treated controls. The sensitivity of P114 and D17 cell-lines increased six- and five-fold, respectively. Doxorubicin treatment in XIAP depleted cells increased sensitivity in BDE cells more than eight-fold, whereas P114 and D17 cell-lines showed an increase in sensitivity of three- and five-fold, respectively.

**Conclusion:**

XIAP directed siRNA's have a strong sensitizing effect on TRAIL-reduced cell-viability and a smaller but significant effect with the DNA damaging drug doxorubicin. The increase in efficacy of chemotherapeutics with XIAP depletion provides the rationale for the use of XIAP siRNA's in insensitive canine tumors.

## Background

The inhibitor of apoptosis proteins (IAPs) are a family of structurally related proteins with anti-apoptotic functions. To date, eight family members have been identified all carrying a functional baculovirus IAP repeat (BIR) domain. Members of the IAP family include Survivin, c-IAP1, c-IAP2, and X-linked inhibitor of apoptosis (XIAP) which directly bind and inhibit caspases 3, 7, and 9 [1]. XIAP (hILP/MIHA/BIRC4) is the most potent caspase inhibitor of all family members [2,3]. XAF1 and Smac/DIABLO regulate XIAP activity, which indicates an important function of this protein in maintaining proper apoptotic functions within the cell.

Apoptosis can be initiated via the intrinsic and/or the extrinsic pathway [4]. The intrinsic pathway is activated by intracellular stress such as growth factor withdrawal, hypoxia, and DNA damage. In this pathway the caspase cascade is triggered by cytochrome c release from the mitochondria. On the other hand the extrinsic apoptotic pathway is triggered by death receptors such as Fas/CD95, TNF receptor, and the TRAIL receptor. Activation of these death receptors usually involves caspase 8 activation which in turn activates effector caspases-3 and -7 [5,6]. Overall, activation of these two pathways is not distinctly separated as activation of one usually involves the other.

Resistance to apoptosis is a hallmark of various (canine) cancers [5]. Indeed in various (chemoresitant) tumors the XIAP protein has been shown to be induced when compared to normal tissue [7-9]. XIAP knock-out mice have shown that the absence of XIAP does not have a negative effect on the development of normal tissues [10]. On the other hand, down-regulation of XIAP with antisense techniques provides antitumor activity in non-small-cell lung cancer (NSCLC) xenografts [11]. Furthermore studies with stable expression of short-hairpin RNAs (shRNA) against XIAP dramatically increased sensitivity of cell-lines to chemotherapies [12]. Thus, XIAP may represent a novel and tumor-selective therapeutic target for anticancer drug design [13].

In this study we describe the use of siRNA's directed against XIAP for sensitizing canine cell-lines to TRAIL and doxorubicin reduced cell-viability. Whereas TRAIL treatment will provide proof of principle, sensitizing tumor cells to doxorubicin will greatly benefit the use of this chemotherapeutical in canine tumors. We chose three cell-lines derived from bile duct-, mammary-, and bone tumor-tissue which could provide the basis for the therapeutic use of siRNA's. Taken together, in order develop anti-neoplastic therapeutic protocols in dog tumors, which represent good clinical models [14], we investigated the effect of XIAP siRNA on different apoptotic agents in canine tumor cell-lines.

## Results

### Establishing a XIAP down-regulation

**Figure 1:**
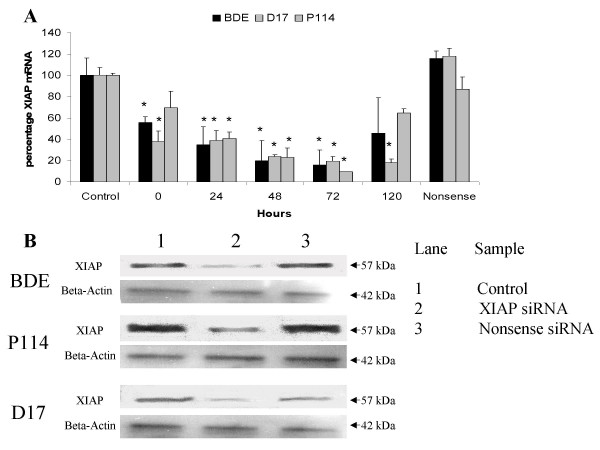
**Down-regulation of XIAP levels compared to control**. Relative mRNA levels from different time points from just after transfection up to 5 days set towards control is shown in (**A**). Data represent mean ± SD of six independent samples (n = 6). Statistically significant differences in down-regulation of XIAP mRNA at different time-points towards control were determined by a student *t*-test (*P < 0.05). Western blot analysis of immunoreactive bands of 57 kDa large XIAP protein levels after 72 hours is shown in (**B**).

Using the Lipofectamine reagent in combination with magnetic assisted transfection (MATra), we transfected the cell-lines with siRNA designed from the canine XIAP gene sequence. The cellular uptake of oligoribonucleotides was initially determined using fluorescent labeled dsRNA (Invitrogen). The transfection-efficiency was optimized and proved to be greater than 95% of the cells as displayed by green fluorescence FITC labeled siRNA (data not shown). When treated with the optimal amount of siRNA (50 nM), the XIAP expression was markedly reduced in all cell-lines with the highest decrease at 72 hours (Figure 1). At this time point the mRNA-levels were decreased to 16 percent in BDE cells, 19 percent in D17 cell-lines, and 9 percent in P114 cell-lines lines, compared to control. At 72 hours Western blotting (Figure 1B) yielded a 57 kDa immunoreactive band of XIAP in the control and nonsense siRNA transfected samples. Specificity was proven in controls without first antibody, which was deemed negative (not shown). Densitometric analysis indicated a strong reduction of XIAP protein in the XIAP siRNA treated samples. Cells treated with nonsense siRNA exhibited a similar expression pattern as the control cells (Figure 1). Viability assays (MTT) indicated an equal cellular homeostasis in controls towards XIAP down-regulated cells in all three cell-lines (data not shown).

### Treatments

**Figure 2:**
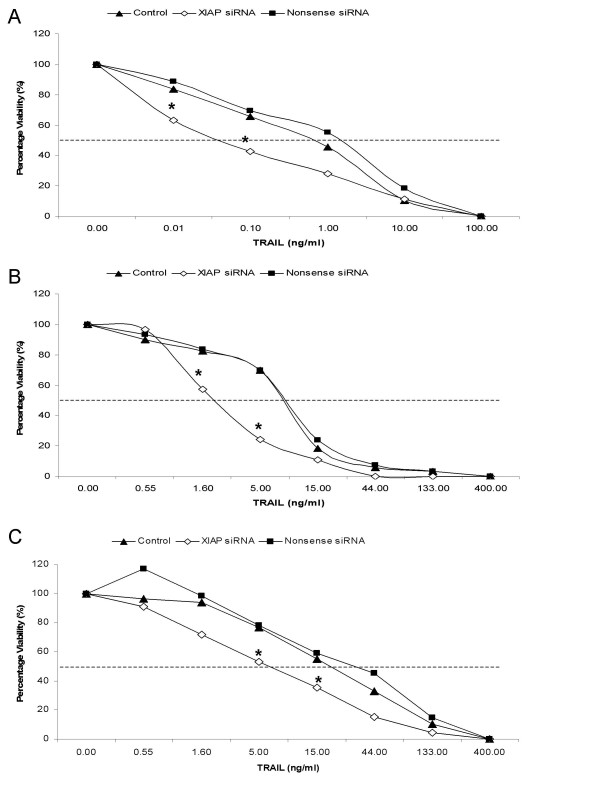
**Effect of XIAP loss on TRAIL sensitivity in canine cell-lines**. The effect on viability is shown for BDE cells in (**A**), for P114 cells in (**B**), and for D17 cells in (**C**). Control (▲); 50 nM XIAP siRNA (◇); 50 nM Nonsense siRNA (■). Data is represented as percentage viability towards untreated control. Points represent average of four independent experiments (n = 4). Statistical significance of differences in viability of the XIAP siRNA treated cell-lines at different drug concentrations compared to control and nonsense were determined by an one-way ANOVA using the Dunnett multiple comparisons test (*P < 0.05).

**Figure 3:**
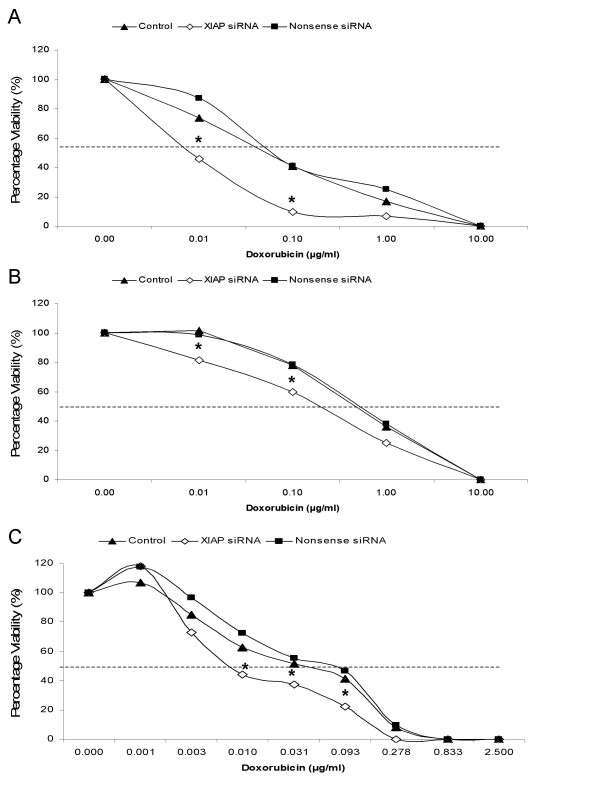
**Effect of XIAP loss on Doxorubicin sensitivity in canine cell-lines**. The effect on viability is shown for BDE cells in (**A**), for P114 cells in (**B**), and for D17 cells in (**C**). Control (▲); 50 nM XIAP siRNA (◇); 50 nM Nonsense siRNA (■). Data is represented as percentage viability towards untreated control. Points represent average of four independent experiments (n = 4). significance of differences in viability of the XIAP siRNA treated cell-lines at different drug concentrations compared to control and nonsense were determined by an one-way ANOVA using the Dunnett multiple comparisons test (*P < 0.05).

We have measured the effect of XIAP depletion by siRNA-mediated gene silencing on TRAIL and doxorubicin induced cell death. As shown in Figure 2, siRNA transfection increased the sensitivity for TRAIL of all three cell-lines used. This effect was highest in BDE cells, whereas P114 cells and D17 were less sensitized by siRNA treatment. XIAP-depleted BDE cells (Figure 2A) showed the strongest increase in TRAIL-sensitivity; a 14-fold reduction of the ED_50_. The P114 and D17 cell-lines showed an increase in sensitivity of six and five-fold, respectively (Figures 2B and 2C). For doxorubicin treatment (Figure 3), again the largest increase in sensitivity was seen in the BDE cells showing a more than eight-fold reduction in the ED_50_. The P114 and D17 cell-lines showed an increase in sensitivity of three and five-fold, respectively (Figures 3B and 3C).

### Gene expression of IAP family members and cellular homeostasis

**Figure 4:**
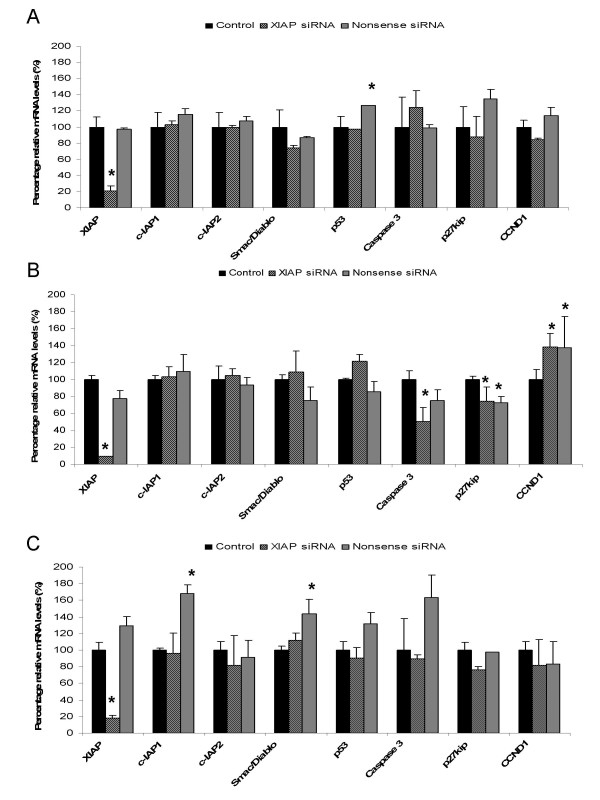
**Gene-expression profiles canine cell-lines**. Quantitative mRNA measurement of IAP family members and gene-products involved in cellular homeostasis. Gene-expression profile of BDE-cells is shown in (**A**), P114-cells is shown in (**B**), and D17-cells is shown in (**C**). Data represent mean ± SD of six independent experiments (n = 6). Statistically significant differences in gene-expression of different treatments towards control were determined by a student *t*-test (*P < 0.05).

In Figure 4, the effect of XIAP siRNA on XIAP, cIAP-1, and cIAP-2 mRNA levels is depicted. Results showed that none of these IAP-family members, other than XIAP, were affected. XIAP mRNA levels were decreased down to 20 percent in BDE cells, 15 percent in P114 cells, and 20 percent in D17 cells. Nonsense siRNA did induce c-IAP1 levels (165 percent) and Smac/Diablo levels (142 percent) in D17 cells. Measurement of parameters for cellular homeostasis revealed an increase in p53 levels in BDE cells and D17 cells in the nonsense siRNA treated cells. The introduction of siRNA's in P114 cells seemed to induce cell-cycle and increase viability through inductions in CCND1, decreases in p27kip, and decreases in caspase-3 mRNA levels in XIAP and nonsense siRNA treated cells.

## Discussion

In the present study we observed a strong decrease of TRAIL- and doxorubicin-induced cell viability with XIAP siRNA in canine cell-lines. We have used three different tumor cell-lines in order to be able to conclude about the general applicability of the outcomes of this study. The first cell-line studied, a canine liver epithelial cell-line of biliary origin (BDE) can be considered as an *in vitro *counterpart of cholangiocarcinoma. The P114 cell-line was derived from a mammary tumor [15], and the third cell-line was derived from a canine osteosarcoma (D17). In order to show the proof of principle that XIAP depletion in canine cell-lines decreases the sensitivity to anti-neoplastic drugs, a treatment with TRAIL was used. As TRAIL solely induces apoptosis through the extrinsic pathway, an increase in sensitivity will show a decreased capacity of inhibiting caspase-3 and -7. Second doxorubicin, a DNA damaging drug which type is frequently being used for treatment of canine tumors, was used.

Although we did not see an reduction of cell-viability in our cell-lines when XIAP was down-regulated (data not shown), as described by several papers [16,17], we could demonstrate an increase in the sensitivity for different chemotherapeutic treatments. The fact that the introduction of XIAP siRNA's alone does not seem to reduce cell-viability indicates a need for triggering the cells by chemotherapy. XIAP has been proposed as a possible treatment of various cancers [18]. Our results showed a strong increase in sensitivity of all three tumor cell-lines to TRAIL treatment up to fourteen fold in XIAP siRNA treated BDE cells. This indicates that the measured results represent a general effect. The fact that all canine cell-lines were increasingly sensitized through XIAP depletion holds promises that therapeutic low concentrations TRAIL may be effectively used without effecting somatic cells.

Doxorubicin hydrochloride, a cytotoxic anthracycline antibiotic, is commonly used in veterinary clinical treatments for various cancers [19]. However, many dog tumors appear to be resistant to doxorubicin. In the *in vitro *experiments of Macy et al. [20], five out of twenty-one carcinomas were deemed sensitive (24 %), whereas three out of thirteen (23 %) sarcomas showed sensitivity to a 14 day treatment with 1 μg/ml doxorubicin. Because of this insensitivity to high amounts of doxorubicin, a pretreatment with XIAP siRNA could be beneficial for many canine tumors, lowering the concentrations needed for a beneficial effect in sensitive tumors and could even make insensitive tumors treatable. Although this added effect remains to be proven *in vivo*, the application of siRNA's against XIAP in combination with a chemotherapeutic agent such as doxorubicin seems a realistic option based on the present results. *In vivo *experiments with siRNA's already have been used in various models [21-24]. In these experiments results show that injected siRNA's are stable and can be found in the blood for long periods, down-regulating long half-life proteins over several days [25]. However, the use of targeted drug-delivery systems should still be considered for siRNA delivery [26] or chemotherapy [27], which further enhances specificity and potentially reduces drug-associated side-effects.

Measurements on gene expression of IAP family members and cellular homeostasis genes did not reveal major differences in cell-cycle progression. In the P114 cell-line, however, the introduction of siRNA's in general seemed to induce the cell-cycle and cellular viability. Although this may imply an adverse effect *in vivo *the MTT measurements did not reveal differences in viability of the groups (data not shown). The IAP family members under study (besides XIAP) did not show many significant differences in gene-expression. In D17 cells however, a significant increase in c-IAP1 and Smac/Diablo was seen in the nonsense-treated cells. The effect of these differences remains elusive, but the antagonistic properties of these proteins could explain why little if any effect was observed in cell viability. Taken together, some differences were seen in cellular homeostasis or IAP family members although the majority indicated an absence of off-target effects. However, unpublished results on TRAIL-treated cells did show differences in gene-expression in c-IAP1. When treated for 5 hours with 50 ng/ml TRAIL, c-IAP1 induced two to three-fold in the D17 cell-line and six to seven-fold in the P114 cell-line, whereas no effect was seen in the BDE cells. This indicates that the reduction in XIAP mRNA when treated with TRAIL, could be counteracted with an increase in c-IAP1. Furthermore, the cell-line with the highest induction in c-IAP1 (seven-fold increase in P114) has the least sensitivity for TRAIL treatment. This result corroborates the hypothesis as described by Harlin et al. where XIAP knock-out mice did not show any negative side effects during development [10]. In this paper no compensation due to increased gene-expression of other family members such as c-IAP1 was seen in untreated cells with XIAP depletion.

The use of siRNA's directed against XIAP to increase chemosensitivity of tumors has been described in several papers [28-30]. Furthermore, clinical trials have started with antisense oligonucleotides to treat solid tumors and hematologic malignancies by Aegera pharmaceuticals (Montreal, Canada) [31]. This study not only corroborates the found results in these papers, the use of osteosarcoma and breast carcinoma derived cell lines could indicate other fields of interest. Concerns mentioned in these papers indicate the possible role of other IAP family members after the targeting of XIAP[32]. As mentioned previously, in the tumor cell lines chosen here, no compensatory effect was seen in the IAP family members indicating a possible positive outcome in the treatment of these tumors.

The use of spontaneously occurring tumors in companion animals as models for human cancer has already been described previously [14]. In this study several tumor cell lines have been suggested as models that offer the best comparative interest, including canine osteosarcoma and mammary tumors. Canine mammary tumour, canine osteosarcoma and possibly also canine hepatocellularcarcinoma [33] resemble human pathologies at the molecular level. Especially the first two are relatively frequently observed in dogs. Therefore we had chosen these cell lines as *in vitro *models for a-proof-of-principle. These *in vitro *results provide a rational for the use of XIAP inhibition (either with siRNA or other small molecules) in *in vivo *therapies in dogs.

## Conclusion

The use of XIAP siRNA to increase the sensitivity of canine tumors for chemotherapy holds great potential. In this study we showed an increase in sensitivity in canine cell-lines derived from osteosarcoma, mammary carcinoma, and cholangiocarcinoma for TRAIL and Doxorubicin reduced cell-viability. These *in vitro *results provide a rational for the use of XIAP siRNA in *in vivo *therapeutical use in dogs.

## Methods

### Cell-lines

Canine bile duct epithelial (BDE) cells were acquired from the Amsterdam Medical Center, Experimental Liver cell bank (Amsterdam, The Netherlands) [34]. BDE cells were grown in DMEM (Life Technologies, Inc., Invitrogen, Breda, The Netherlands) supplemented with 580 mg/l glutamine, 10 μg/ml gentamicin, and 10% heat-inactivated fetal bovine serum (FBS; Harlan Sera-Lab, Loughborough, United Kingdom) at 37°C in a humidified atmosphere of 5% CO_2 _in air. P114 canine mammary tumor cells were acquired from Dr. Rutteman from the department of clinical sciences of companion animals, faculty of veterinary medicine, Utrecht university, the Netherlands. P114 cells were grown in DMEM:F12 medium (Invitrogen, Breda, The Netherlands) containing 580 mg/l glutamine, 10 μg/ml gentamicin, and 10% FBS at 37°C in a humidified atmosphere of 5% CO_2 _in air [15]. The canine osteosarcoma cells (D17) were acquired from the American Type Culture Collection (ATCC, Cat.no. CRL-6248) and were maintained in DMEM medium (Invitrogen) supplemented with 580 mg/l glutamine, 10 μg/ml gentamicin, and 10% FBS at 37°C in a humidified atmosphere of 5% CO_2 _in air. For all experiments cells were seeded in a concentration of 4 × 10^3 ^cells per well in a 96-well plate 24 hours before transfection.

### Establishment of the down-regulation of XIAP in canine tumor cell-lines

For silencing experiments, Stealth™ dsRNA molecules were obtained from Invitrogen. A specific sequence for canine XIAP silencing (5'-CCAUGUGCUAUACAGUCAUUACUUU-3') was selected after general recommendations (Invitrogen) and was designed from the canine XIAP gene sequence (Genbank accession no. AY603038). A nonsense sequence was used as a negative control (5'-GCAGGUGCUAGUACAAGUCCGACAA-3'). Transfection was performed with the Magnet Assisted Transfection (MATra) technique (IBA BioTAGnology/Westburg b.v., Leusden, The Netherlands), in combination with lipofectamine2000™ (Invitrogen), according to the manufacturer's instructions. In short, 50 nM siRNA molecules were transfected into the cell-lines in the presence of an optimized concentration Lipofectamin2000™ (1.2 μl/ml), for 20 minutes on the plate magnet under cell-culture conditions. After transfection, growth media including antibiotics replaced the transfection media. Control samples were mock transfected with lipofectamine2000™ and magnetic beads from the MATra technique.

### Treatments

For TRAIL treatment, a serial dilution of 400 to 0 ng/ml recombinant TRAIL (R&D Systems Europe Ltd., Abingdon, United Kingdom) was used in DMEM media (Invitrogen) including 10 % FCS, glutamine (580 mg/l), and gentamycin (10 μg/ml). Treatment started 48 hours after transfection. After a 24 hour treatment proliferation and viability was measured with a MTT assay (5 mg/ml). For doxorubicin treatment, a serial dilution of 100 to 0 μg/ml doxorubicin hydrochloride (Pharmachemie b.v., Haarlem, The Netherlands) was used diluted in growth medium. Treatment started 48 hours after transfection. After 24 hour treatment proliferation and viability was measured with a MTT assay (5 mg/ml). The experiment was repeated four times, averages of the four independent experiments were used in the analysis. Statistical significance of differences in viability of the XIAP siRNA treated cell-lines at different drug concentrations compared to control cells were determined by an one-way ANOVA using the Dunnett multiple comparisons test. P < 0.05 was considered to indicate statistical significance. Analysis was performed using SPSS software (SPSS Benelux, Gorinchem, the Netherlands).

### RNA isolation and Reverse-transcription polymerase chain reaction

For each group (control, XIAP siRNA, or nonsense siRNA) the RNA of six independent experiments was isolated. Total cellular RNA was isolated with the Qiagen RNeasy Mini Kit according to the manufacturer's instructions (Qiagen, Leusden, The Netherlands). In short, RNA was isolated from each sample by adding 100 μl lysis buffer (RLT containing 1 % (v/v) β-mercaptoethanol) directly after decanting the media. The RNA samples were treated with DNase-I (Qiagen RNase-free DNase kit). In total 3 μg of RNA were incubated with poly(dT) primers at 42°C for 45 min, in a 60 μl reaction volume, using the Reverse Transcription System from Promega (Promega Benelux, Leiden, The Netherlands).

### Quantitative measurements of the mRNA levels

Quantitative real-time PCR was performed on a total of 10 gene products; GAPDH, HPRT, XIAP, c-IAP1, c-IAP2, Smac/Diablo, p53, caspase-3, p27kip, and CCND1. Technical triplicates were measured for each cDNA in the real-time-PCR analysis. The abundance of mRNA was measured by real-time quantitative PCR using appropriate primers (Table 1) as previously described [35]. In short, Q-PCR was based on the high affinity double-stranded DNA-binding dye SYBR^® ^green I. For each experimental sample, the amount of the gene of interest and of the two independent endogenous references (glyceraldehyde-3-phosphate dehydrogenase (GAPDH) and hypoxanthine phosphoribosyl transferase (HPRT)) was determined from the appropriate standard curve in autonomous experiments. If relative amounts of GAPDH and HPRT were constant for a sample, data were considered valid and the average amount was included in the study (data not shown). Results were normalized according to the average amount of the endogenous references. The normalized values were divided by the normalized values of the calibrator (healthy group) to generate relative expression levels [36]. A Kolmogorov-Smirnov test was performed to establish a normal distribution and a Levene's test for the homogeneity of variances. If samples were normally distributed, the statistical significance of differences between diseased and control animals was determined by using the Student's *t*-test. A p-value < 0.05 was considered statistically significant. Analysis was performed using SPSS software (SPSS Benelux, Gorinchem, the Netherlands).

**Table 1:** Nucleotide Sequences of Canine Specific Primers for Real-Time Quantitative PCR.

Gene	Primer	Sequence (5'-3')	Tm (°C)	Product size (bp)	Accession number
GAPDH	Forward	TGT CCC CAC CCC CAA TGT ATC	58	100	AB038240
	Reversed	CTC CGA TGC CTG CTT CAC TAC CTT			
HPRT	Forward	AGC TTG CTG GTG AAA AGG AC	56	100	L77488/L77489
	Reversed	TTA TAG TCA AGG GCA TAT CC			
XIAP	Forward	ACT ATG TAT CAC TTG AGG CTC TGG TTT C	54	80	AY603038
	Reversed	AGT CTG GCT TGA TTC ATC TTG TGT ATG			
c-IAP1	Forward	AGG CGT CCC CGT GTC CGA GAG	68	96	XM_858260
	Reversed	TAG CAT CAG GCC GCA GCA GAA GC			
c-IAP2	Forward	AGG CCA ATG TAA TTA ATA AAC AGG A	62	94	DQ223014
	Reversed	AAC TAA GAC AGT ATC AAT CAG TTC TCT C			
Smac/DIABLO	Forward	AGC AGA AGC TGC ATA TCA AAC TGG AG	62	90	XM_534661
	Reversed	ACT TCC TGC ACC TGC GAC TTC AC			
p53	Forward	GCC CCT CCT CAG CAT CTC ATC	67	100	NM_001003210
	Reversed	GGC TCA TAA GGC ACC ACC ACA C			
Caspase 3	Forward	ATC ACT GAA GAT GGA TGG GTT GGT	58	140	AB085580
	Reversed	GAA AGG AGC ATG TTC TGA AGT AGC ACT			
Bcl-2	Forward	TGG AGA GCG TCA ACC GGG AGA TGT	61	87	AB116145
	Reversed	AGG TGT GCA GAT GCC GGT TCA GGT			
p27KIP	Forward	CGG AGG GAC GCC AAA CAG G	60	90	AY455798
	Reversed	GTC CCG GGT CAA CTC TTC GTG			
CCND1	Forward	ACT ACC TGA ACC GCT	56	151	AY620434
	Reversed	CGG ATG GAG TTG TCA			

### Western blot analysis

Samples were homogenized in 350 μl RIPA buffer containing 1 % Igepal, 0.6 mM Phenylmethylsulfonyl fluoride, 17 μg/ml aprotinine and 1 mM sodium orthovanadate (Sigma chemical Co., Zwijndrecht, The Netherlands) for 30 minutes on ice. Protein concentrations were obtained using a Lowry-based assay (DC Protein Assay, BioRad, Veenendaal, The Netherlands). Fifteen μg of protein of the supernatant were denatured for 3 min at 95°C and electroforesed on 15 % Tris-HCl polyacrylamide gels (BioRad) and the proteins were transferred onto Hybond-C Extra Nitrocellulose membranes (Amersham Biosciences Europe, Roosendaal, The Netherlands) using a Mini Trans-Blot^® ^Cell blot-apparatus (BioRad). The procedure for immunodetection was based on an ECL Western blot analysis system, performed according to the manufacturer's instructions (Amersham Biosciences Europe). The membranes were incubated with 4 % ECL blocking solution and 0.1 % Tween 20 (Boom B.V., Meppel, The Netherlands) in TBS for 1 hour under gentle shaking. Primary antibodies were incubated at 4°C overnight. For XIAP a mouse anti-dog XIAP (BD Biosciences, Alphen aan den Rijn, The Netherlands) was used in a dilution of 1:1,000 in TBST with 4% BSA. As a loading control a mouse anti-dog Beta-Actin antibody was used in a 1:2,000 dilution in TBST with 4% BSA. After washing, the membranes were incubated with a goat anti-mouse (R&D Systems/Westburg b.v.) in TBST with 4% BSA for 1 hour at room temperature. Exposures were made with Kodak BioMax Light-1 films (Sigma chemical Co.).

## Competing interests

The author(s) declare that they have no competing interests.

## Authors' contributions

BS made the experimental plan, designed siRNAs, performed Q-PCR measurements, and wrote the manuscript. MJ performed cell culture studies, transfections, viability assays, and helped to interpret the data. BA helped perform the Western blot experiments and helped in drafting of the manuscript. GR delivered the cell lines and helped setup the initial studies. JR revised the manuscript. LP participated in the design of the study, assisted in coordination of the work, and helped draft the manuscript. All authors have read and approved the final manuscript.
